# Surfactant replacement therapy in preterm infants with congenital heart disease: Physiological concepts and therapeutic considerations

**DOI:** 10.1038/s41372-026-02654-5

**Published:** 2026-04-20

**Authors:** Arvind Sehgal, Patrick J. McNamara, Samuel Menahem

**Affiliations:** 1 Monash Children’s Hospital, Monash University, Melbourne, VIC Australia; 2 Department of Paediatrics, Monash University, Melbourne, VIC Australia; 3 Department of Paediatrics, University of Iowa, Iowa City, IA USA; 4 Division of Neonatology, University of Iowa Stead Family Children’s Hospital, Iowa City, IA USA; 5 Paediatric and Fetal Cardiac Units, Monash Medical Centre, Monash Health, Melbourne, VIC Australia

**Keywords:** Cardiovascular diseases, Outcomes research

## Abstract

A prevalence rate of 8.8/1000 live-births for congenital heart disease has been reported. The clinical outlook of these infants is dependent on transition from intra-uterine life to postnatal life. Preterm infants are also commonly administered surfactant to manage respiratory distress syndrome. This physiology-based narrative describes optimal oxygen saturation, mechanical ventilation practices and circulatory imbalances that might happen after surfactant administration in preterm infants with congenital heart disease. Clinicians may consider higher oxygen requirement thresholds for surfactant therapy in this select cohort, in comparison to infants without congenital heart disease. The interplay between surfactant deficiency, surfactant replacement therapy, and the unique interaction with underlying congenital heart disease represents a knowledge gap. This perspectives article discusses the haemodynamic vulnerability of preterm infants and discusses the circulatory impact of surfactant in premature infants without and with structural heart disease. We provide physiology-based suggestions for duct-dependent lesions, parallel circulations and other structural heart disease.

## Introduction

The clinical outlook of infants with congenital heart disease (CHD) has improved due to ongoing advances in maternal-fetal medicine care, neonatology, fetal imaging-paediatric cardiology and peri-operative cardiac care. Timely and accurate pathophysiology guided management, attention to possible secondary organ damage, and specific input from multi-disciplinary sub-specialities are crucial in optimizing outcomes. A prevalence rate for CHD of 8.8/1000 live-births has been previously reported in data from the National Institute of Child Health and Human Development Perinatal Collaborative Project which incorporated >56,000 deliveries [1]. In the Baltimore-Washington Infant regional epidemiologic study, the prevalence rate of CHD was 3.7/1000 livebirths [2]. More recent estimates indicate that to be about 5 to 8/1000 live newborns; half of these defects are serious and account for approximately 30% of infant deaths [3]. In a recent study from Kids Inpatient Database, serious CHDs were noted to have a greater prevalence as well as in-hospital mortality in premature infants compared to term infants (7.4/1000 births vs. 1.5/ 1000 term births, *P* < 0.001) [4]. An international cohort study from 2007–2015 for premature very low birthweight infants ranging between 24 and 31 weeks’ gestational age (GA) compared in-hospital mortality and morbidity outcomes in 609 (0.77%) infants with serious CHD compared with 76,371 preterm infants without any cardiac malformation [5]. The mortality rate was higher in the former (18.6% vs. 8.9%), with increased odds for chronic lung disease (CLD), necessitating careful consideration of respiratory strategies and interventions. Evidently, this cohort may require unique considerations for optimal management due to their maturational haemodynamics [5–7].

While respiratory distress syndrome (RDS) may contribute to poor oxygenation, the optimal oxygen saturations and a routine approach to mechanical ventilation for preterm infants with CHD, especially related to physiology secondary to surfactant replacement therapy (SRT) is not well-defined. The interplay between surfactant deficiency, SRT, and the unique interaction with CHD represents a knowledge gap. The objectives of this narrative are to discuss the haemodynamic vulnerability of preterm infants and discuss the circulatory impact of SRT in premature infants without and with structural heart disease. While the questions related to oxygenation of premature infants with congenital heart defects and the circulatory response to SRT have not been studied systematically, we endeavor to provide physiology-based suggestions.

## Surfactant and its effects on neonatal survival and morbidities

Five, oftentimes overlapping stages characterize lung development. These include (a) embryonic, (b) pseudoglandular, (c) canalicular, (d) saccular, and (e) alveolar. The canalicular stage is highlighted by alveolar cellular differentiation into type 1 and type 2 pneumocytes, of which the latter is responsible for surfactant production. Alveologenesis involves the establishment of a large gas exchange surface area, and the maturation of type 2 pneumocytes and surfactant synthesis. Improved clinical outcomes of infants with RDS after SRT are aided by an increase in lung compliance [8–10]. A systematic review of 13 randomized controlled trials for SRT noted significant improvements in oxygenation, ventilation requirements and reduction of air leak, mortality before hospital discharge, and death or CLD at 28 days, compared with placebo [11]. Essentially, the unequivocal benefits of SRT in the modern neonatal intensive care are well-established [9, 12–17].

## Unique aspects of the preterm cardiovascular system

Preterm infants need individualized care due to the immaturity of various organ systems; these include the lungs (surfactant deficiency) and the heart (immature myocardium with under-developed contractile apparatus). SRT for RDS has a dramatic impact on lung recruitment, gas exchange and blood flow patterns, and therefore may have major haemodynamic consequences in the setting of major CHD. Two main aspects of the normal transitional circulation are relevant to the altered cardiovascular physiology of CHD; namely closure of the patent ductus arteriosus (PDA) and the fall in pulmonary vascular resistance (PVR). In premature infants, the PDA frequently stays patent for longer, thereby modulating blood flow between the systemic and pulmonary circulations. In addition, unlike in the healthy term infants, the PVR remains elevated in the setting of RDS, contributing to inadequate aeration of the lung and poor pulmonary blood flow (PBF), leading to hypercapnia and hypoxemia [18]. The transition from intra-uterine life also involves two changes in cardiac loading conditions. *First*, left heart preload is augmented due to an increase in PBF after lung aeration. *Second*, right atrial preload transiently decreases secondary to cord-clamping. In essence, both sides of the heart are affected by transitional changes [19, 20]. The developmental vulnerability of the immature myocardium, used to contracting against a low-resistance placental circulation *in utero*, places preterm infants at an increased risk of impaired ventricular function and circulatory impairment.

## Surfactant replacement therapy in infants with structurally normal heart

SRT is known to influence the balance between the magnitude of PDA shunt and the PVR/PBF indices, in both animal and human experiments [21–30]. In 2022, nearly a quarter [2876, (23.2%)] of Australian and New Zealand Neonatal Network registrants were administered exogenous surfactant for RDS. Of these, 44.2% received multiple doses [31]. Recent data has detailed the effects of SRT on transitional neonatal haemodynamics [28–30]. Previously published literature on haemodynamic effects of SRT in cohorts without CHD is briefly summarized below.

Evidence from preterm lambs using radioactive microsphere injections noted increased ductal flow and a decline in PVR in response to SRT with natural surfactant [23]. *Poractant alpha* (derived from natural porcine lung surfactant) had a small but significant dose-dependent dilating effect in isolated mouse PDA, putatively due to nitric oxide signaling (relaxant effect) [32]. Experiments in newborn piglets with surfactant deficiency noted that the vasodilatory effect of porcine surfactant is associated with activation of nitric oxide synthase [33]. Data in these cohorts has s also noted dose-dependent vasodilatory effects of modified porcine lung surfactant (Curosurf), which was accompanied by effects on cardiac output [34]. In mechanically ventilated infants who received synthetic surfactant, increased PBF and magnitude of left to right PDA shunt was noted [24, 25]. A significant and rapid fall in pulmonary arterial pressure, coinciding with a fall in oxygen requirements after SRT has also been noted with synthetic surfactant [24–27]. Natural SRT (*bovine lipid extract surfactant*) also resulted in a decrease in PVR and increased PBF [28]. Essentially, the direct/indirect impact of SRT on PVR can promote a major haemodynamic shift, favoring the pulmonary circulation (at the expense of systemic circulation) through the PDA [30].

SRT was also accompanied by a drop in LV output and an increase in the RV: LV output ratio [28]. Investigators have shown either no change or decreased LV output following SRT, likely secondary to increased left to right atrial shunting [28, 35, 36]. The clinical impact of altered systemic blood flow is equally important due to the fragility of the cerebral circulation and its vulnerability to rapidly fluctuating systemic blood flow. A recent study of infants with GA 28 weeks noted a significant increase in systolic blood pressure (BP), and drop in diastolic BP, although the mean arterial BP was unchanged [28]. Hence, it is important to be mindful of the fluctuations in systemic haemodynamics in response to SRT, due to the potential downstream end-organ effects, which have been recently summarized [37, 38].

## Congenital heart disease: Sentinel haemodynamics & adaptations to SRT

**Fig. 1:**
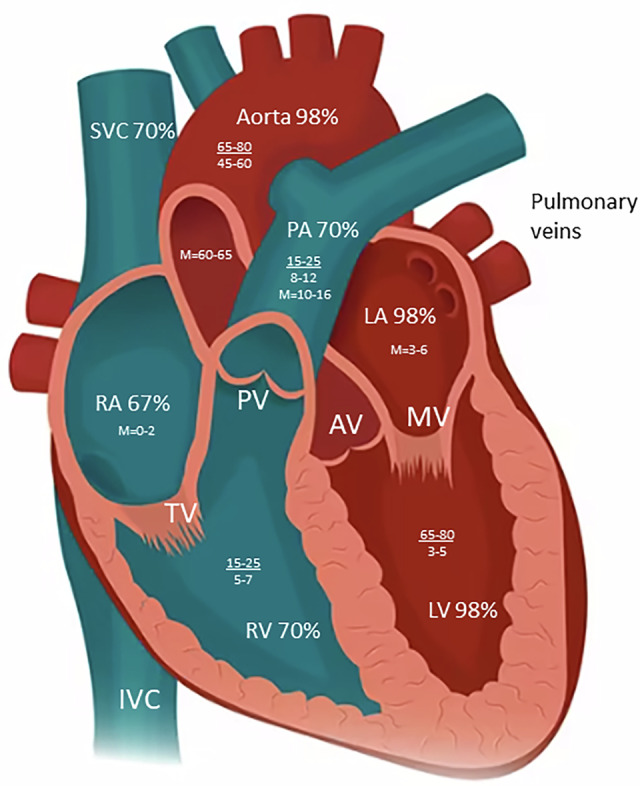
Normal cardiac anatomy. Oxygen saturations values and haemodynamic pressures in respective chambers obtained by cardiac catheterization in a term-newborn infant (after transition from fetal to postnatal physiology [fall in pulmonary vascular resistance, closure of patent ductus arteriosus and no significant shunting through the *foramen ovale*). RA-right atria, RV-right ventricle, LA-left atria, LV-left ventricle, MV- mitral valve, TV-tricuspid valve, PV-pulmonary valve, AV-aortic valve.

**Fig. 2:**
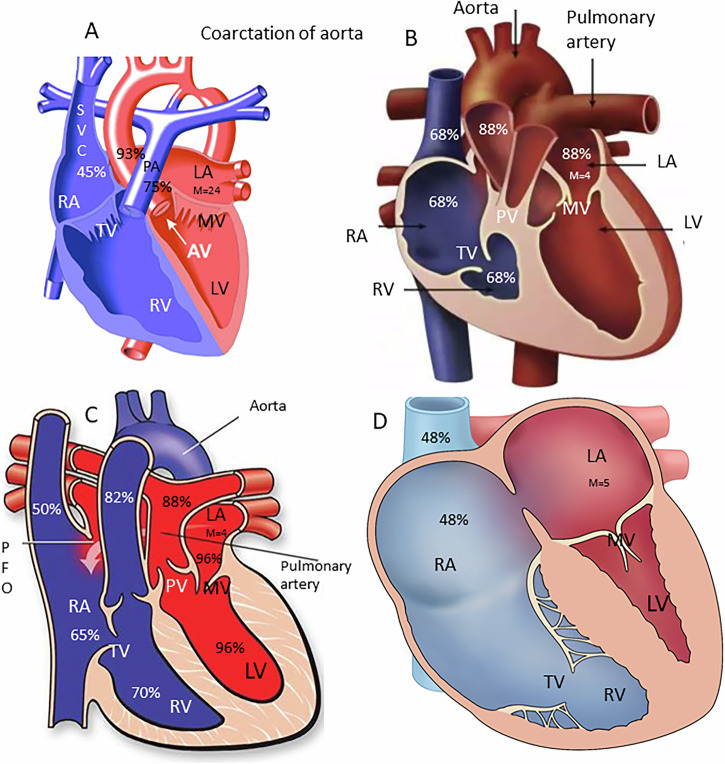
Congenital heart disease haemodynamics: oxygen saturations and mean pressure in respective chambers. M=mean value for pressure in mm Hg. **A** Coarctation of aorta (elevated LA pressure could lead to left to right atrial shunt, and pulmonary edema and secondary pulmonary artery hypertension). **B** Pulmonary atresia with intact ventricular septum (supra-systemic RV pressure with right to left atrial shunting with systemic desaturations). **C** Transposition of great arteries with intact ventricular septum (higher oxygen saturations in the pulmonary artery than aorta, shunting from LA to RA through atrial septal defect [white arrow], large ductus arteriosus may contribute to pulmonary hypertension). **D** Ebstein anomaly (right to left shunting at the atrial level and aortic arterial saturations ~78%, left to right shunt through the PDA leading to pulmonary hypertension, lower LV output reflected in low superior venacava saturations). RA-right atria, RV-right ventricle, LA-left atria, LV-left ventricle, MV- mitral valve, TV-tricuspid valve, PV-pulmonary valve, AV-aortic valve, PFO-patent foramen *ovale*.

Infants with RDS due to surfactant deficiency present with impaired oxygenation and respiratory failure, making it challenging to differentiate clinically whether these symptoms reflect intrinsic lung disease vs. CHD. Clinical effects of management of a premature infant with CHD requiring SRT has been summarized in Table 1, encapsulating the putative circulatory imbalances. CHD states are broadly classified into four categories. In Table 2, we summarize generic physiologic principles relevant to each category (rather than each specific lesion), alongside a very brief description of the relevant CHD. The circulatory influences of SRT are also elucidated. General guiding principles in premature infants with CHD include optimization of lung recruitment while avoiding excessive mean airway pressure. At the same time adequate positive end-expiratory pressure may be required to overcome pulmonary oedema. In our centre, SRT is administered to preterm infants <32 weeks GA if requiring FiO_2_ > 0.3. While it is difficult to prescribe optimum/higher threshold for preterm infants with co-existing CHD, clinicians should individualize therapy keeping in mind the possibility of a sudden drop in PVR and increase in PBF, inducing imbalance between systemic/ pulmonary circulations. An individualized threshold may be determined by joint consultation between neonatologists and cardiologists after a review of chest radiograph and flow-dynamics from echocardiography. Figure 1 depicts normal cardiac anatomy and saturations in a term infant. Figure 2 depicts oxygen saturations and unique aspects in different forms of CHD. Post-ductal oxygen saturations may be lower than the upper body (pre-ductal) saturations; of note, upper body saturations reflect cerebral perfusion. Lower oxygen saturation (75 to 80%) are generally accepted.

**Table 1:** Case summary.

Demographics and delivery room management	Cardiac and lung imaging	Course post-SRT via LISA
-Male, 31 weeks gestation, birthweight 1800g -Mode of delivery: caesarean section due to placenta praevia -Antenatal steroids: 2 doses -CPAP 7 cm of H_2_O and maximum initial FiO_2_ 0.8 to maintain oxygen saturations >70%; gradually ↓ to 0.4 -Hemodynamically stable (mean BP 40 mm Hg) -Blood gas pH 7.24, pCO_2_ 53 mm Hg and base deficit -2.	-Antenatal: Tetraology of Fallot’s -Postnatal: Large malaligned peri-membranous VSD with bidirectional shunt, severe RVOT obstruction (predominantly valvar with additional infundibular and mild supravalvar stenosis) with confluent good sized branch pulmonary arteries, moderate RV hypertrophy and normal biventricular systolic function -Chest radiograph: diffuse granular opacities	-Administered as baseline FiO_2_ > 0.3 (as per Unit guideline) -Within four hours, ↑ in FiO_2_ to 0.55 as saturations remaining below 70% -Hemodynamically unstable (mean BP: 27 mm Hg and blood gas: to pH 7.05, pCO_2_ 93 mm Hg and base deficit -4) -Intubation and conventional mechanical ventilation needing FiO_2_ 0.8 → administered 2^nd^ dose of SRT→ no improvement in oxygenation Blood gas: pH 7.16, pCO_2_ 60 mm Hg and base deficit -8 in FiO_2_ of 1.0 -HFO and inotropic support (noradrenaline) instituted -Subsequent improvement in BP and reduction in FiO_2_ to 0.6

*SRT*surfactant replacement therapy, *LISA*less invasive surfactant administration, *CPAP*continuous positive airway pressure, FiO_2_fraction of inspired oxygen, *RVOT* right ventricular outflow tract, *VSD* ventricular septal defect, *BP* blood pressure, *HFO* high frequency oscillation.

**Table 2:** Circulatory adaptations in congenital heart disease (CHD) and influence of surfactant replacement therapy (SRT).

Category	Example	Duct dependence	Physiologic considerations	Effects of SRT	Physiology-based suggestions
Duct dependent systemic circulations	*Severe aortic stenosis *Coarctation of aorta *Interrupted aortic arch *HLH	Yes	*May be accompanied by LV hypertrophy (except HLH). *LV becomes the sole supplier of systemic output after PDA closure (except HLH). *Back-pressure leading to altered LA end-diastolic pressure.	*Routine SRT at conventional FiO_2_ may lead to a sudden ↓ in PVR and ↑ in PBF may induce imbalance between systemic/ pulmonary circulations.	*Since ↑ FiO_2_ is a strong pulmonary vasodilator, keep FiO_2_ < 0.5 to 0.6 (unless severely hypoxemic). *Routine SRT at conventional FiO_2_ threshold such as 0.3 should be avoided. *Cautious SRT to ↓ FiO_2_ decreases to <0.5 if thought to have severe RDS & and needing >0.6 FiO_2_.
Duct dependent pulmonary circulations	*Severe pulmonary stenosis *Pulmonary atresia with intact septum^ *Tetralogy of Fallot (TOF) *Tricuspid atresia *Severe Ebstein anomaly	Yes (except selected TOFs depending on the degree of RV outflow tract obstruction)	*In pulmonary atresia, the MAPCAs narrow over time, resulting in worsening cyanosis from ↓ PBF. In other cases, the pulmonary arteries may be well formed and confluent. *Coronary artery fistulae connecting with RV cavity may make the LV susceptible to ischemia (hence the circulatory shift towards pulmonary circulation [and away from systemic circulation] may be deleterious). *TOF with absent pulmonary valve may have significant dilatation of the main and proximal branch pulmonary arteries and already ↑ PBF.	(a) Inability to ↑ PBF due to the nature of defect in pulmonary atresia. (b) The negative impact of coronary steal on LV perfusion (ischemia→ ↓ LV compliance→ pulmonary venous congestion→ ↓ lung compliance). (c) In TOF with good-sized branch pulmo(nary arteries), sudden reduction in PVR secondary to SRT may lead to circulatory imbalance. In Ebstein’s anomaly, supportive measures to ↑ PBF and ↓ PVR include ↑ FiO_2_ supplementation & mild respiratory alkalosis.	*Routine SRT is inadvisable. Cautious SRT may ↓ PVR and supplement ductal flow if needing ↑ FiO_2_ > 0.6 *(except if accompanying pulmonary valve insufficiency)*.
Parallel circulation	Transposition of great (TGA) arteries with or without VSD	Yes	*Critical to ensure adequate mixing between the two parallel circulations.	*Controlled hyperventilation to ↓ PVR and ↑ PBF (avoiding pCO_2_ < 30 mm Hg).	*Cautious SRT due to its effects on ↓ *PVR and* ↑ *PBF if needing* ↑ *FiO*_*2*_ > *0.6*.
Other lesions	a. Truncus arteriosus b. Obstructed TAPVC c. Complete atrioventricular defects	No No No	*PBF is increased. *Any intervention to further ↑PBF/ ↓ PVR could predispose to early pulmonary vascular disease. *Severely compromised PBF *Limitation to PBF is not due to limitation in antegrade flow into the pulmonary arteries but due to outflow obstruction at the level of veins. Pulmonary artery pressures are generally at systemic level earlier, especially if there is a large VSD component (more so when accompanied by Trisomy 21).	*SRT may be counterproductive leading to rapid deterioration.	*Surfactant replacement therapy has no role to play. *Cautious SRT early on if* ↑ *PVR and needing high FiO*_*2*_. > *0.6*. *Inadvisable if continued need for* ↑ *FiO*_*2*_ *as high-risk of circulatory imbalance*.

*HLH* hypoplastic left heart, *FiO*_2_ fractional inspired oxygen, *LV* left ventricular, *PEEP* positive end expiratory pressure, *RDS* respiratory distress syndrome, *PDA* patent ductus arteriosus, *PVR* pulmonary vascular resistance, *PBF* pulmonary blood flow, *RV* right ventricle, *MAPCA* major aorto-pulmonary collateral arteries, *VSD* ventricular septal defect, *CPAP* continuous positive airway pressure, *TAPVC* total anomalous pulmonary venous connection, *CHF* congestive heart failure. ^Two distinct varieties- a. reasonable sized pulmonary arteries supplied by PDA *in utero* ± VSD and b. severely hypoplastic pulmonary arteries with MAPCA.

### Duct dependent systemic circulations

The common pathway of these defects is the limitation to systemic blood flow. Specifically, the clinical consequences of systemic hypo-perfusion include hypotension and low cardiac output state (decreased urinary output, elevated lactate and metabolic acidosis). The sudden elevation in LV pressure (as might happen in coarctation of aorta) may lead to impairment in LV contractility. High oxygen delivery may lower PVR leading to an increased systemic to pulmonary shunting through a PDA or VSD that may further exaggerate the compromise to systemic perfusion. On the other hand, a drop in PVR post-SRT may also preferentially direct blood flow towards the pulmonary circulation (at the expense of systemic circulation). The arterial blood gas is quite informative. For instance, in hypoplastic left heart, if oxygen saturations are >90% but are accompanied by metabolic acidosis, it represents a significant imbalance between pulmonary and systemic perfusions. Low oxygen saturation (75 to 80%) accompanied by a normal pH indicate an acceptable balance between pulmonary over-circulation and systemic hypo-perfusion. A lower saturation target (and paO_2_) allows for a higher pCO_2_. Maintaining a relatively high PVR will assist to optimize right to left flow across the PDA.

SRT may be typically inadvisable, however there are patients (usually extremely preterm infants, especially with no antenatal steroid cover) with true RDS that may benefit from SRT. The decision to treat must carefully consider its putative effects in inducing or accentuating a circulatory imbalance. Table 2 summarizes the SRT related haemodynamics and alterations.

### Duct dependent pulmonary circulation

These abnormalities result in a reduction of PBF as the common denominator. Infants can be hypoxemic due to right to left shunting through the PFO, as the pressure in the RV (oftentimes hypertrophic and non-compliant) is often greater than the LV pressure. Often, multiple aorticopulmonary collateral arteries that perfuse segments of the lung coexist (Table 2). Ebstein anomaly on the other hand is characterized by atrialization of the RV, and very limited forward flow into the pulmonary artery.

Optimizing lung recruitment at the same time avoiding excessive mean airway pressure are key, alongside optimizing lung compliance, as this will ensure adequate left-right shunt through the PDA. These infants might benefit from measures to decrease PVR and promote antegrade PBF, and cautious administration of SRT with close haemodynamic monitoring may have a role to play. Table 2 summarizes seminal haemodynamics and SRT related alterations. Especially in Ebstein anomaly, SRT is likely to be helpful in the acute setting; however, once the PVR has fallen, further doses of surfactant are not likely to be helpful and may cause harm. However, caution needs to be exercised if accompanied by pulmonary valve insufficiency as reversal of flow through the PDA (ductal steal) may induce sudden deterioration secondary to systemic under-perfusion [39].

### Parallel circulation

Transposition of the great arteries is characterized by a parallel circulation such that systemic venous return is pumped through the aorta back to the systemic circulation and the pulmonary venous return being pumped through the pulmonary artery back into the pulmonary circulation. Therefore, maintenance of shunts through the PFO or PDA by septostomy and prostaglandin infusion are essential. In transposition, if the patient needs a left-right PDA shunt in the presence of an ongoing restrictive atrial communication, cautious SRT administration with close haemodynamic monitoring may be utilized (Table 2).

### Other congenital heart diseases

Table 2 summarizes circulatory changes in these lesions. The archetypal example of obligatory mixing is the truncus arteriosus. Most of these infants present with symptoms of congestive heart failure early in the neonatal period because of already substantially increased PBF. Total Anomalous Pulmonary Venus Connection (TAPVC) involves drainage of the pulmonary veins into the systemic venous circulation. Complete mixing of the pulmonary and systemic venous return occurs. Frequently, the infra-diaphragmatic drainage may get obstructed, leading to pulmonary venous hypertension and pulmonary edema, and a chest radiograph that may be difficult to confidently distinguish from RDS. In the presence of severely restricted PFO and obstruction to the pulmonary venous flow, severe hypoxemia, respiratory distress and cyanosis are obvious. Complete atrioventricular defects most commonly take the form of a common single atrio-ventricular valve. Trisomy 21 is a common accompanying diagnosis and PVR frequently stays elevated for prolonged period.

In obstructed anomalous veins, measures such as SRT to reduce PVR and increase PBF can lead to sudden development of or worsening of pre-existing pulmonary edema, leading to rapid clinical deterioration. Routine SRT should be avoided. In case of prenatally undiagnosed anomalous veins in a preterm infant, in case SRT is administered based on chest radiograph suggesting RDS, rapid deterioration could ensue, and an urgent echocardiogram must be arranged. Lung ultrasound is being increasingly used to diagnose RDS [40]. This opens up exciting possibilities wherein RDS could be differentiated from TAPVC associated lung edema. Literature is currently lacking, possibly due to the rarity of the diagnosis, but needs prospective multi-centric evaluation. Due to elevated PVR in atrioventricular defects, overtime giving way to pulmonary over-circulation, therapy mainly involves adequate respiratory support. SRT may be used very cautiously in the acute/early stage but runs the risk of rapidly tipping the circulation towards a significantly increased PBF. Table 2 summarizes the essential haemodynamics and SRT related alterations.

### Clinical consequences emanating from circulatory imbalances

The clinical circulatory impact of SRT for RDS remains under-appreciated. The timing of clinical presentation and the accompanying symptomatology depends on three variables: (a) the nature and severity of the anatomic defect, (b) *in utero* haemodynamic consequences arising from the heart defect (such as congestive heart failure/hydrops) and (c) the interplay between the structural lesion and the impact of the transitional circulation. The consequent effects on cardiovascular physiology are likely to be accentuated in the setting of prematurity.

The direct/indirect impact of SRT on PVR may promote a major haemodynamic shift. These effects may be exaggerated in patients with a PDA and lead to excessive PBF at the expense of systemic circulation [30]. SRT is also accompanied by a drop in LV output and an increase in the RV: LV output ratio [28]. The consequences of these haemodynamic shifts may be further exaggerated in patients with duct-dependent systemic circulations, due to the fragility of the cerebral circulation and its vulnerability to rapidly fluctuating systemic blood flow [41]. A recent study of infants with GA 28 weeks and weighing approximately 1300 g noted significant variations in systolic BP [28]. These effects are important as coronary artery perfusion pressure depends on diastolic BP. Reduced myocardial perfusion leading to myocardial fatigue is undesirable in patients with CHD. Hence, it is important to be mindful of the fluctuations in systemic haemodynamics in response to SRT, due to the potential downstream end-organ effects, which might reflect biochemically as increased serum lactate levels.

## Summary

In summary, the decision to administer SRT in premature infants with CHD is challenging and must weigh the benefits of optimization of lung recruitment and minimization of the risks of major air leaks with negative effects on pulmonary vs. systemic haemodynamics. Essential to the decision to administer SRT is confirmation of the diagnosis of RDS, which has classic radiologic features on chest radiographs or lung ultrasound [40], so as to avoid exposure of patients without RDS to the adverse effects of non-judicious SRT use. It is important to recognize the fact that the potential benefits of SRT may be greater in patients with duct-dependant pulmonary defects where left-right flow is lifesaving. Given the challenges of the decision to administer SRT, communication between neonatologists and paediatric cardiologists around this topic is advisable. In addition, there exist many gaps in the scientific understanding. Supplementary Table S1 enlists knowledge gaps and research priorities in the management of premature infants with CHDs. We encourage clinicians and researchers actively involved in the care of this high-risk population to appraise this practice scientifically and develop innovative research to enhance our knowledge.

## Supplementary information

Supplementary Table legend S1

Supplementary Table S1
