# Supplementary material for: Surfactant replacement therapy in preterm infants with congenital heart disease: Physiological concepts and therapeutic considerations
Source: J Perinatol. 2026 Apr 20;46(7):1350–6. doi: 10.1038/s41372-026-02654-5 (PMC13423785; doi:10.1038/s41372-026-02654-5)
Supplement: Supplementary file 1 — Supplementary Table legend S1 [file 41372_2026_2654_MOESM1_ESM.docx]

**Supplementary table legend**

Supplementary Table S1: Knowledge gaps and research priorities
