# Supplementary material for: Surfactant replacement therapy in preterm infants with congenital heart disease: Physiological concepts and therapeutic considerations
Source: J Perinatol. 2026 Apr 20;46(7):1350–6. doi: 10.1038/s41372-026-02654-5 (PMC13423785; doi:10.1038/s41372-026-02654-5)
Supplement: Supplementary file 2 — Supplementary Table S1 [file 41372_2026_2654_MOESM2_ESM.docx]

Supplementary Table S1: Knowledge gaps and research priorities

| 1. Need for physiologic/pharmacologic studies of SRT in preterm infants with CHD. Sub-group analysis should include gestation-specific categorization such as ≤24 weeks, 25-28 weeks, 28-31 weeks and > 31 weeks gestational age. 2. Phenotypic characterization of information related to response to SRT in CHD infants, by grouping according to disease physiology (systemic vs pulmonary duct dependence). 3. Network-wide data collection as to the incidence of SRT administration in this cohort. 4. Specifically documenting a comparison of oxygenation and haemodynamic response to SRT. 5. Knowledge of the interdependence of the heart and lungs is essential for neonatal intensivists and paediatric cardiologists/ surgeons caring for this cohort. 6. To systematically evaluate the response to SRT by using comprehensive, accurate and objective haemodynamic multi-modality monitoring using echocardiography, Near Infra-Red Spectroscopy, non-invasive cardiac output monitoring and amplitude-integrated electroencephalogram (38). |
| --- |

CHD-congenital heart disease, SRT-surfactant replacement therapy
